# Applications of Hydrogels with Special Physical Properties in Biomedicine

**DOI:** 10.3390/polym11091420

**Published:** 2019-08-29

**Authors:** Gong Chen, Wenwei Tang, Xiaohui Wang, Xueling Zhao, Cheng Chen, Zhigang Zhu

**Affiliations:** 1 School of Environmental and Materials Engineering, College of Engineering, Shanghai Polytechnic University, Shanghai 201209, China; 2 Modern Service Department, College of International Vocational Education, Shanghai Polytechnic University, Shanghai 201209, China; 3 Research Center of Resource Recycling Science and Engineering, Shanghai Polytechnic University, Shanghai 201209, China

**Keywords:** hydrogel, physical properties, biomedicine

## Abstract

As a polymer matrix containing a large amount of water, hydrogels have been widely used in many fields such as biology and medicine due to its similarity to extracellular matrix components, and its contact with blood, body fluids, and human tissue does not affect the metabolic processes of living organisms. However, due to the lack of unique physical properties of traditional polymer hydrogels, its further application in the high-end field is limited. With the progress of study, a series of hydrogels with special structures, such as double network hydrogel, composite hydrogel, Tetra-PEG gel, and topological gel, have improved the situation to a large extent. At the same time, the progress of research on the biocompatibility and biodegradability of hydrogels, which are expected to be used in biomedical fields, is also worthy of attention. This review introduces four such types of high-strength polymeric hydrogels and the mechanisms for improving their mechanical strength. Moreover, a discussion will be made around specific methods for imparting special physical properties to hydrogels and applications in the field of biomedicine such as cell culture, medical surgery, tissue engineering, and biosensing. At the end of the review, the main reasons and contradictions for the limits of the current applications are explained. An outlook on the future research in related fields and the importance of carrying out research in this area to promote medical progress are emphasized.

## 1. Introduction

Hydrogels are polymer network systems containing a large amount of water in the matrix [1] with water as a dispersion medium, and they are insoluble in water due to the existence of a special crosslinked structure. At the same time, the physical and chemical properties of hydrogels are very similar to most human tissues, which are composed of polysaccharides and proteins [2]. Not only do hydrogels generally not affect the metabolic processes of the living body, they also allow metabolites to be discharged through them. Compared with any other synthetic biological materials, hydrogels are similar to living tissue and are similar in nature to the extracellular matrix components in particular, providing a suitable environment for cell survival and growth. Moreover, hydrogels can reduce the friction and mechanical effects on the surrounding tissue after water absorption. Thus, hydrogels with biocompatibility, degradability, and controllable mechanical properties have broad application prospects in biological fields such as tissue engineering and 3D cell culture [3]. Hydrogels currently used biomedical applications include natural polymers [4,5,6], synthetic polymer hydrogels [7], and natural/synthetic polymer composite hydrogels [8]. Under normal circumstances, the polymer concentration of the hydrogel is positively correlated with its mechanical properties, and this also means lower biocompatibility and degradability. This contradiction limits the use of hydrogels in biomedical applications [9,10]. It is well known that the brittleness of hydrogels is mainly derived from irregular structures, and the design of a series of high-strength polymer hydrogels with regular or special network structures has become the strategy toward solving this problem.

**Figure 1:**
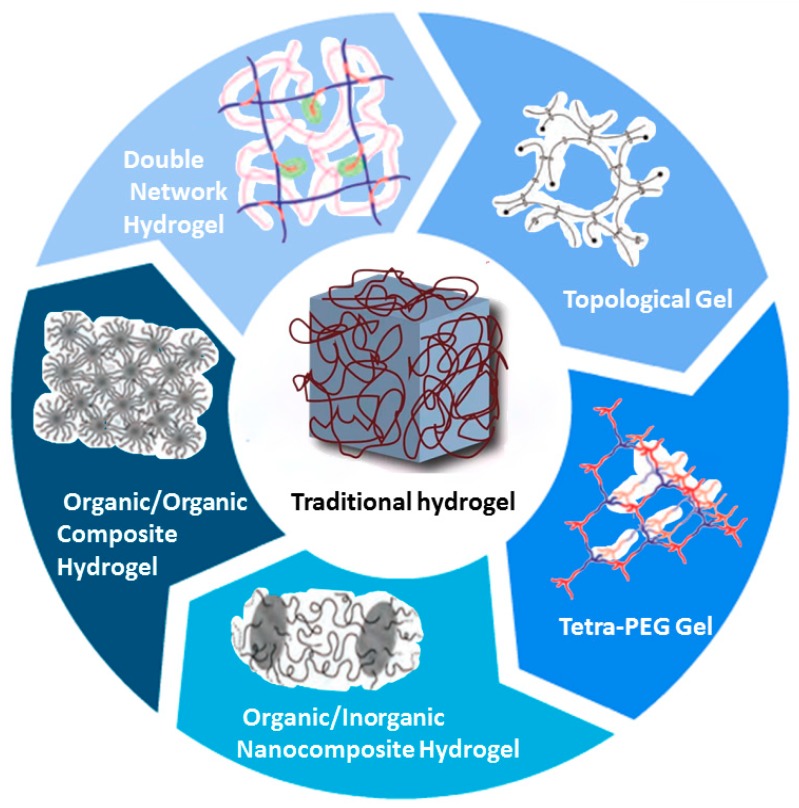
Typical network structures of traditional and several high-strength hydrogels [12].

In recent years, with the development of hydrogels with excellent physical properties [11], new applications have been obtained in the field of biomedicine, and advances in medicine have been promoted. This paper introduces four common hydrogels with regular morphology and mechanical properties: double network hydrogels, composite hydrogels, tetra-(polyethylene glycol) (Tetra-PEG) gels, and topological gels. The application of hydrogels with special physical properties built in different methods in cell culture, medical surgery, tissue engineering, biosensing, and so on, are also discussed [Figure 1].

## 2. Polymeric Hydrogels with Mechanical Properties

### 2.1. Double Network Hydrogel

**Figure 2:**
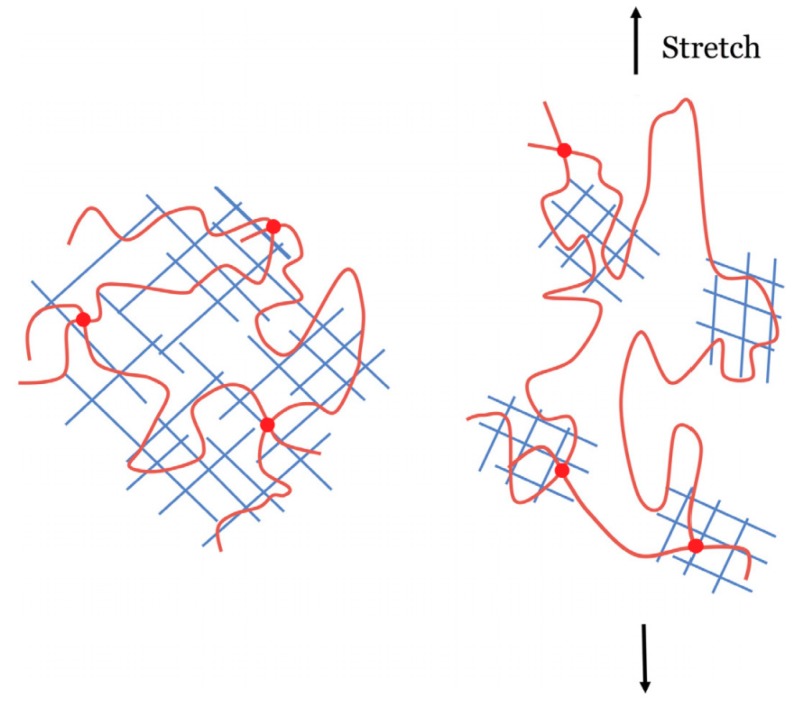
A double-network hydrogel consists of two polymer networks: a short-chain network that can swell nearly to the breaking point, and a long-chain network that is highly stretchable. When the hydrogel is stretched, the short-chain network ruptures, but the long-chain network remains elastic [18].

Double network (DN) hydrogels were proposed by Professor Gong and her collaborators at Hokkaido University in 2003 [13]. The DN is a multi-purpose functional system that consists of two interpenetrating polymer networks [14,15]. The first layer is a highly crosslinked rigid polymer short chain network polymer, the second layer is a slightly crosslinked or non-crosslinked flexible polymer long chain network entangled through the first network, and these two networks are interconnected [16,17]. As shown in Figure 2, when external force is applied, the rigid, brittle network can sacrifice a part of the first break and dissipate energy to prevent greater breakage while the long chain network maintains the elasticity and shape of the gel, and the two networks synergize together such that the hydrogel exhibits excellent mechanical properties [18].

### 2.2. Composite Hydrogel

#### 2.2.1. The Organic/Inorganic Nanocomposite Hydrogel

The organic/inorganic nanocomposite hydrogel is the nanocomposite material formed by dispersing an inorganic phase on nanometer scale into an organic polymer matrix. Nanocomposite (NC) gels have the strength and thermal stability of inorganic materials as well as the functionality of organic polymers [19,20]. The mechanism of NC in improving mechanical strength is similar to that of rubber reinforcing [21], i.e., by using the surface activity of carbon black particles to adsorb the molecular chains of rubber, forming similar physical crosslinkages so as to play a powerful role in reinforcement and greatly improve its mechanical properties. NCs, depending on the types of nanoparticles, can be divided into the following categories: clay composite hydrogels [22], polymer particle composite hydrogels [23], carbon nanotube reinforced polymer hydrogels [24], and crosslinked polyvinyl alcohol (PVA) nanofiber hydrogels [25].

#### 2.2.2. The Organic/Organic Composite Hydrogel

**Figure 3:**
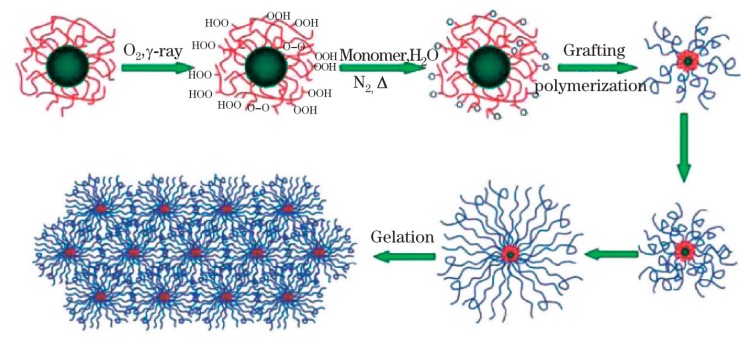
Proposed mechanism for formation of macromolecular microsphere composite (MMC) hydrogel and MMC hydrogel microstructure [28].

The organic/organic composite hydrogel is represented by the macromolecular microsphere composite (MMC) gel [26], which uses rigid inorganic nanoparticles or macromolecular microspheres as single and multiple crosslinking centers [27]. The mechanisms for the formation of the peroxide and the initiation of polymerization, as well as for the formation of MMC gel, are proposed in Figure 3. MMCs are mainly composed of MMSs (macromolecular microspheres), chains, and water molecules [28]. The polymer chains are connected to each other, and when subjected to an external force, the uniform polymer chain length and the crosslinking can effectively absorb and dissipate the center of energy. The hydrogel thus demonstrates excellent mechanical properties.

### 2.3. Tetra-PEG Gel

In 2008, Sakai [29] developed a four-arm polyethylene glycol (PEG) gel. Tetra-PEG is a tetrahedral polyethylene glycol macromonomer modified by two symmetrical end groups, tetra-amine-terminated PEG (TAPEG) and tetra-N-hydroxysuccinimide-glutarate-terminated PEG (TNPEG), which presents as a tetrahedral structure [30]. The results of small angle neutron scattering (SANS) showed that there was virtually no spatial inhomogeneity [31]. In addition, unevenness does not occur even under balanced expansion conditions [32]. Thus, entire networks can be approximately considered as a single network, and this is also the source of its excellent mechanical properties. Due to its mechanical properties and biocompatibility, Tetra-PEG gels can be used as a potential biomedical material [33].

### 2.4. Topological Gel

The topological gel is also called the Slide-Ring Gel, which was synthesized by Okumura and Ito in 2001 [24]. Topological gels are obtained by crosslinking cyclodextrin on different chains of polyrotaxane based on PEG/α-cyclodextrin [34,35]. The difference in microstructure from traditional hydrogel is the random crosslinking points formed by the topological gel can, which slide along the segment while stretched by an external force, essentially acting like a “pulley” (As shown in Table 1). It can evenly divide the long chain into several short chains so that the force is evenly distributed to each segment [36,37].

## 3. Research Focus

### 3.1. Biocompatibility

Biocompatibility refers to the property of living tissue to react with inactive materials [52,53]. Hydrogels implanted in the body should have good biocompatibility due to their direct contact with tissues and cells. Biocompatibility studies of hydrogel materials have been ongoing since the discovery of the excellent biological properties of p-hydroxyethyl methacrylate (HEMA) by Wichterle and Lim in the 1960s [54]. Biocompatibility can be divided into two parts: biological reaction and material reaction. Biological reactions include blood reactions, immune reactions, and tissue reactions; and material reaction mainly manifests in the change of physical and chemical properties of the materials.

In 1948, Rosen first reported the use of murine fibroblast cultures to screen for polymers, and began the research and application of cytotoxicity experiments to evaluate the biocompatibility of materials [55]. The earliest and most widely used cells in the current cytotoxicity test were strain L cells, which are fibroblasts isolated from the subcutaneous tissue of mice in 1948 by Earle [56]. Another cell type that is more widely used is the uterine mucosal cell (HeLa cells) isolated from human uterine tumors in 1953 [57]. Thanks to the advantage of easy passage, rapid reproduction, approachable in vitro culture conditions, and easy storage, these two established cell lines can provide stable passage cells for experiments and be shared by many materials for cytotoxicity evaluation. In 1982, L929 cells and HeLa cells were recommended as standard cells in cytotoxicity assays by the American Society for Quality (ASQ). However, the immune response and tissue repair processes in the body are complex, and it is not sufficient to determine the biocompatibility of a material through cells or tissues.

### 3.2. Biodegradability

Biodegradable polymer materials refer to materials that can be degraded or metabolized by a living organism under certain conditions. Such materials may be involved in human metabolism and eventually pass out of the body [58]. Given that the hydrogel material in tissue engineering is expected to have a certain mechanical strength in addition to having excellent biocompatibility, biodegradability research is expected to achieve gel self-degradation in vivo in order to avoid multiple surgeries for patients. In addition, drug-loadable degradable hydrogels can be sustained in the body for several days or even weeks, thus greatly improving drug availability and patient tolerance, and reducing drug side effects [59]. If a stimuli-responsive element such as an acid, an enzyme, a light, or a heat is introduced into the hydrogel material, the hydrogel drug carrier can be further imparted with more flexible drug-controlled release properties. Currently, biodegradable hydrogel materials are sodium alginate, cellulose, polylactic acid, polyamino acids, and synthetic microorganisms [60,61].

Furthermore, when these hydrogels undergo partial degradation by the tissue, it is possible to produce inherent toxic monomers or oligomers. Even if the polymer may not be toxic, its monomers or shorter oligomers, including dimers [62], trimers [63], and so on, would illicit harmful toxicity. In short, partial degradation of these monomers and shorter oligomers can cause further kidney, heart, and liver damage [64]. For example, PEG is non-toxic [65], but ethylene glycol monomers are toxic to many tissues [66]. In the process of enzymatic hydrolysis and conversion, and the conjugation of these monomers or shorter oligomers to glycine or glucuronic acid, the released organic and inorganic nanoparticles can pass through the blood-brain barrier (BBB) [67] to damage the brain. Some products formed by enzymatic degradation in tissues may not be easily excreted from the kidneys, so accumulation problems would soon occur.

## 4. Applications

### 4.1. Cell Culture

**Figure 4:**
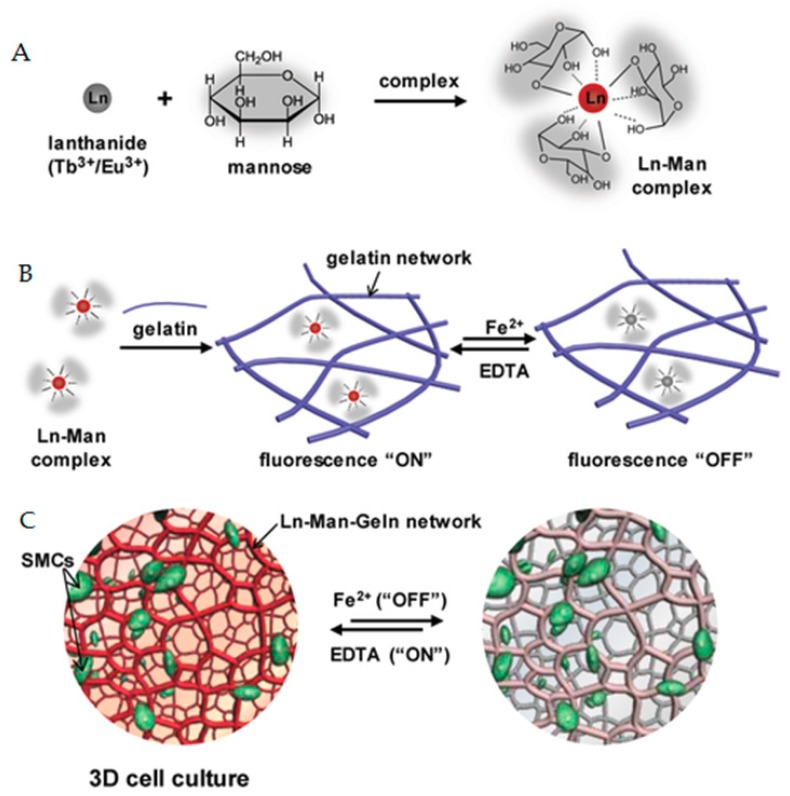
A schematic diagram of the preparation of a reversibly responsive fluorochromic hydrogel based on a lanthanide–mannose complex. (**A**) Scheme of the formation of lanthanide–mannose (Ln–Man) complex through the coordination of mannose with lanthanide ions (Tb^3+^/Eu^3+^). (**B**) Scheme of the formation of lanthanide–mannose–gelatin (Ln–Man–Geln) hydrogel by introducing Ln–Man into the gelatin network, and the property of fluorescence “On/Off” upon addition of Fe^2+^/EDTA. (**C**) Illustration of the Ln–Man–Geln hydrogel as a 3D cell culture matrix for a reversible fluorochromic “On/Off” switch upon addition of Fe^2+^/EDTA [73].

Research on cellular biomaterials in the last decade has turned toward three-dimensional approaches [68]. Hydrogels provide a good 3D cell culture scaffold by simulating the natural extracellular environment. Hydrogels such as VitroGel and GelMA (c.f. Section 4.2 Medical Surgery) have been proven to be non-cytotoxic in many ways and are therefore widely used in 3D cell culture [69,70,71,72]. In order to provide an effective observation strategy for three-dimensional culture of multicolored fluorescent staining cells, Meng prepared a rare-earth-mannose-based hydrogel with a reversible fluorescence color response behavior [73]. The study used mannose as a ligand to act on rare earth ions in aqueous solution to sensitize rare earth ions. A gelatin network was introduced into the system to successfully prepare rare earth hydrogels. Figure 4 shows that the obtained rare earth hydrogel has a reversible fluorescent discoloration behavior in response to external metal ions. Because the hydrogel has good biocompatibility, it is used as a three-dimensional culture medium for cells, and the fluorescence signal of the matrix hydrogel can be controlled into “on” and “off” states during the observation process of the cells, which is involves multicolor fluorescent stained cells. Three-dimensional culture provides an effective observation strategy.

More interestingly, Wilkinson designed a hematopoietic stem cell culture system that uses PVA in culture fluids to culture hematopoietic stem cells using high levels of thrombopoietin and low levels of stem cell factors, and the hematopoietic stem cells of the experimental mice proliferated extensively [74]. PVA is a major component in some common glues. It has been reported that Japanese researchers have experimented with ordinary glues that can be purchased at convenience stores and found that good results have also been obtained for the cultivation of hematopoietic stem cells.

### 4.2. Medical Surgery

**Figure 5:**
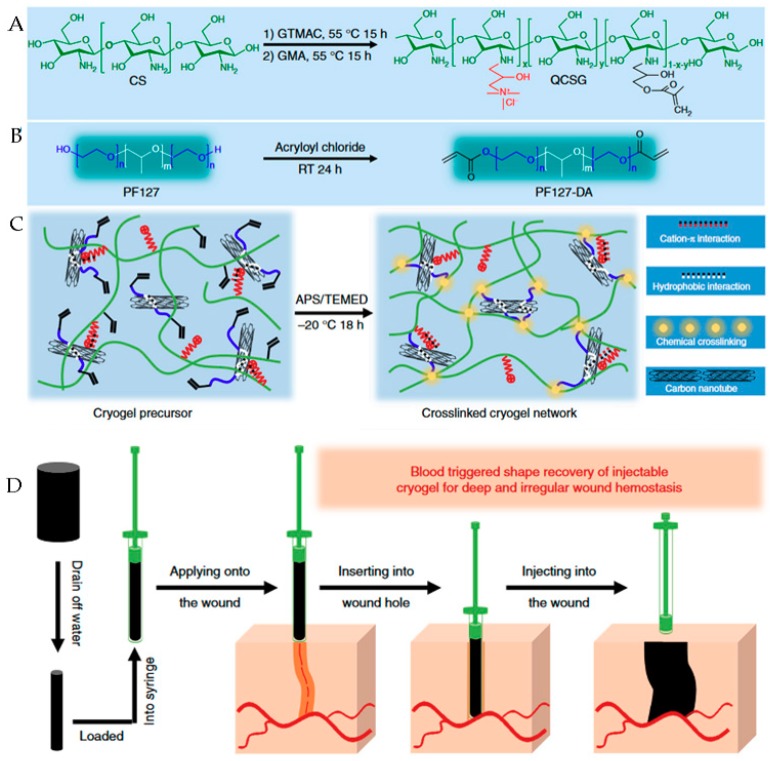
Schematic representation of glycidyl methacrylate functionalized quaternized chitosan/carbon nanotube (QCSG/CNT) cryogel synthesis and application. (**A**) Synthesis of QCSG copolymer. (**B**) Synthesis of PF127-DA copolymer. (**C**) Preparation of QCSG/CNT cryogel. (**D**) Schematic representation of the hemostatic application of injectable shape memory cryogel hemostatics in a deep and irregularly shaped wound mode; the shape memory cryogel could be injected into the narrow, deep, and irregular wound in a shape-fixed state, and they would then immediately absorb and concentrate the blood, and instantly recover their initial volume to fill the irregular wound site while keeping robust mechanical strength [75].

Uncontrolled bleeding results in more than 30% of traumatic deaths, more than half of which occur before emergency care arrives. For deep wounds in particular, it is difficult to stop bleeding by pressure or a tourniquet due to the irregular shape of the wound, and currently, the hemostasis commonly employed in surgery does not show satisfactory effects on deep wounds. Zhao used chitosan derivatives and carbon nanotube materials to prepare injectable shape memory nanocomposite porous colloidal hemostatic materials, shown in Figure 5 [75]. As the main skeleton of the crystal gel, chitosan derivatives provide good hemostatic properties and promote wound healing and blood-triggered shape memory recovery. As a nanofiller, the carbon nanotube material can improve the mechanical properties of the gel, and the carbon nanomaterial can further improve the procoagulant performance of the composite gel. The composite cell’s blood cell and platelet adsorption and activation capabilities allow it to exhibit excellent hemostatic properties in a variety of deep lesions. The shape memory composite gelatin hemostatic dressing has low raw material cost, is easy to prepare, and has great application potential in deep wound hemostasis.

**Figure 6:**
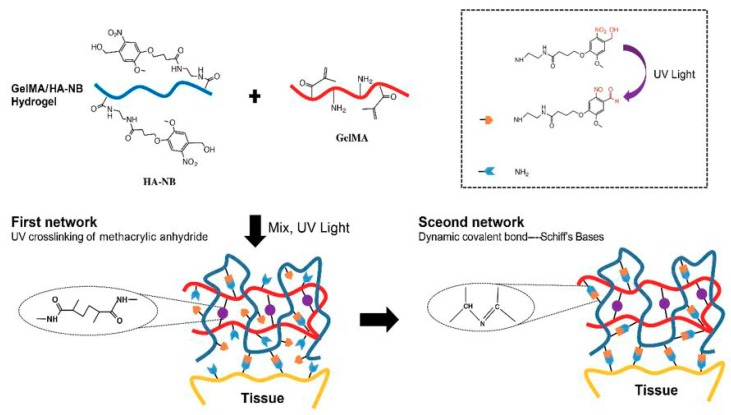
Constituent chemical structures and a schematic diagram illustrating the formation of the photo-triggered imine-crosslinked matrix hydrogel. The effective wet-adhesion properties of the matrix gel derive from photo-generated aldehyde groups bonding with the amino groups on the tissue surface and its mechanical strength derives from its two internal covalently crosslinked networks. Upon UV irradiation, the methyl methacrylate-modified gelatin (GelMA) rapidly generates the first crosslinked network elements of the hydrogel. Meanwhile, some of the photo-generated aldehyde groups on the o-nitrobenzyl-based modified hyaluronic acid (HA-NB) at the tissue–hydrogel interface react with amino groups of the tissue proteins and proteoglycans, forming Schiff bases. Meanwhile, the photo-generated aldehyde groups on the hydrogel HA-NB react with the amino groups of GelMA to form a second crosslinking network. As the reaction progresses, the remaining aldehyde groups react with amino groups of the tissue and GelMA to bond tissue and increase internal crosslinking. The creation of two different chemically crosslinked matrices lead to a significant increase in the tissue adhesion and internal strength of the hydrogel. The good wet tissue adhesion and mechanical properties of the hydrogel would ensure its stability in wound closure [76,79].

Furthermore, rapid hemostasis and the realization of wet side hemostasis are also huge clinical needs. At present, there is no effective rapid hemostasis for arterial and major organ bleeding. As shown in Figure 6, Hong [76] carried out the crosslinking technique with human tissue material as a template based on Yang’s work [77]. By modifying the natural protein and polysaccharide, the biomimetic and wet tissue adhesion was obtained with strong mechanical properties. The main components of the matrix gel are methyl methacrylate-modified gelatin (GelMA) and a o-nitrobenzyl-based light trigger molecule (NB) modified hyaluronic acid (HA-NB). After illumination, the double bonds on the GelMA are self-bonded to form a first layer network. The photogenerated aldehyde groups on the HA-NB are linked to the amino groups on the GelMA to form a second layer network, which greatly enhances the mechanics of the colloid. At the interface between the colloid and the tissue, the photo-aldehyde group of HA-NB reacts with the amino group on the tissue, which can effectively enhance the adhesion of the colloid on the tissue, thereby completely stopping the aortic injury or heart-penetrating injury within a few seconds.

On the other hand, the methacrylic acid (MA) graft substitution conversion rate is the core parameter used to measure the properties of GelMA. When the substitution rate is higher than 15%, it can be photo-cured with crosslinking, and the substitution rate reaches up to about 90%. When the substitution rate is low, GelMA exhibits superior biocompatibility. However, in this process, if the grafting is incomplete or not subjected to dialysis, the toxic small molecule MA may remain [78], and contact with the wound may lead to further inflammatory reactions. These are potential risks that cannot be ignored in the field of medical surgery.

### 4.3. Tissue Engineering

**Figure 7:**
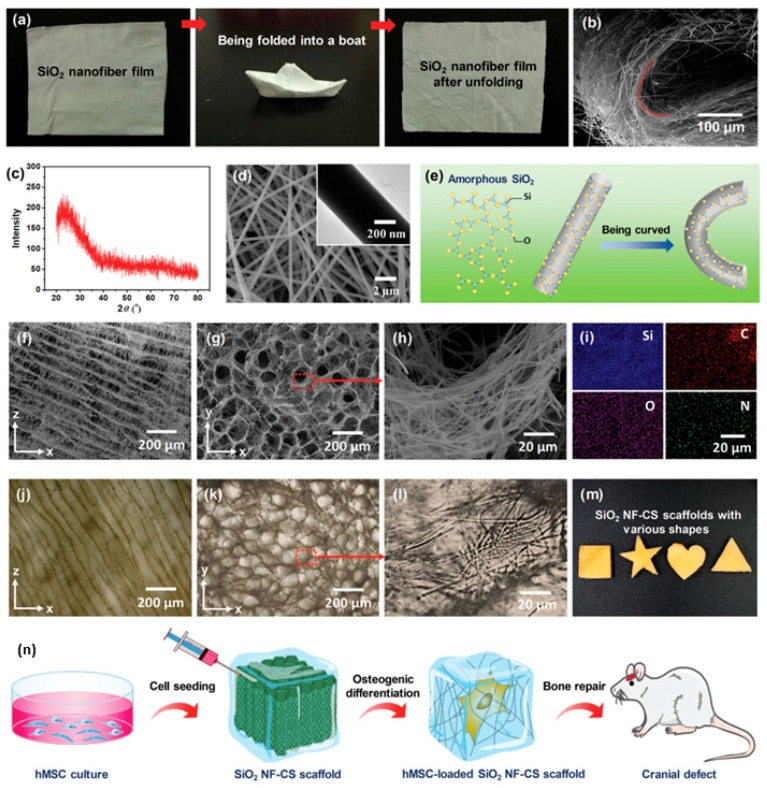
Construction of 3D nanofbrous scaffolds from flexible inorganic nanofbers. (**a**) Flexible SiO_2_ nanofbers with foldability. (**b**) SEM image of a curved SiO_2_ nanofbrous mat showing that SiO_2_ nanofbers could achieve 180° deflection without fracture. (**c**) X-ray diffractometer spectrum indicating amorphous state of SiO_2_ nanofbers. (**d**) SEM image of SiO_2_ nanofbers showing flawless surface. (**e**) Schematic of molecular motion when SiO_2_ nanofbers curved. (**f**) Longitudinal-section and (**g**) cross-section SEM images showing SiO_2_ NF-CS scaffolds possessed honeycomb-like structure. (**h**) SEM image of pore wall consisting nanofber networks to imitate native ECM. (**i**) Elemental maps suggesting that chitosan homogeneously covered on SiO_2_ nanofbers. (**j**–**l**) Optical microscope images of hydrated SiO_2_ NF-CS scaffold showing maintained cellular architecture and nanofbrous networks in aqueous medium. (**m**) Photograph of SiO_2_ NF-CS scaffolds with various shapes. (**n**) Culture of hMSC in SiO_2_ NF-CS scaffolds [80]. In the field of tissue engineering, cartilage occupies a very special position. Due to the lack of blood supply, the ability of cartilage to recruit cells when it receives damage is far weaker than other tissues in the body [81]. Therefore, the construction of cartilage substitute materials with certain mechanical strength, biocompatibility, and cell adhesion is of great significance for clinical cartilage repair.

Irregular bone defects, or bone defects such as cartilage, tendons, and ligaments, are a clinical problem in the treatment of bone defects. Autologous bone grafting is still the first choice for bone repair, but the limited source of autologous bone and the tendency of lesion formation in the donor site severely limit its application. Therefore, tissue engineering scaffolds that facilitate minimally invasive surgery and adaptive irregular bone defects and promote bone regeneration are expected to be ideal bone repair materials. As illustrated in Figure 7, Wang prepared a flexible SiO_2_ nanofiber membrane by utilizing a sol-gel electro-spinning method and further prepared a super-elastic SiO_2_ nanofiber-shell polymer by homogenous dispersion freeze-drying of the flexible SiO_2_ nanofiber composite chitosan solution [80]. The stent can fully recover to its initial height and porous structure under an 80% strain cycling compression in a water environment, and it has a fast recovery rate and good fatigue resistance. The SiO_2_ nanofiber-chitosan (NF-CS) scaffold can be implanted into the rabbit mandibular defect area of different shapes under compression; it quickly recovers the initial shape after absorbing body fluids, and it closely fits to the bone. The preparation method of the stent is simple and flexible. By adjusting the injection ratio of SiO_2_ nanofibers and chitosan, a scaffold with a morphological structure and a mechanical property gradient distribution is prepared, and the biomineralization and mesenchymal stem cell differentiation gradient distribution is realized in soft tissue. It has broad application prospects in bone defect repair at the interface.

Surprisingly, PVA hydrogels exhibit properties comparable to cartilage in many respects [82,83,84], and further research on PVA has become a hot research topic in cartilage tissue engineering [85]. Zhou prepared methacrylated polyvinyl alcohol (PVA-GMA) as the first step [86]. On this basis, photo-crosslinking PVA-GMA/HAP (hydroxyapatite) nanocomposite hydrogels were prepared. Photo-crosslinking PVA-GMA/HAP nanocomposite hydrogels have cell compatibility with cells and can promote cell proliferation. By adding HAP nanoparticles, nanocomposite hydrogels have enhanced mechanical strength and cell adhesion, which shows their potential as scaffolds for tissue engineering.

Meanwhile, although the above hydrogel has very low cytotoxicity, the process of degradation in the body does not rule out the production of toxic molecules and further damage to the body caused by entering the blood circulation system. The specific mechanism and solution still need further research.

### 4.4. Biosensing

**Figure 8:**
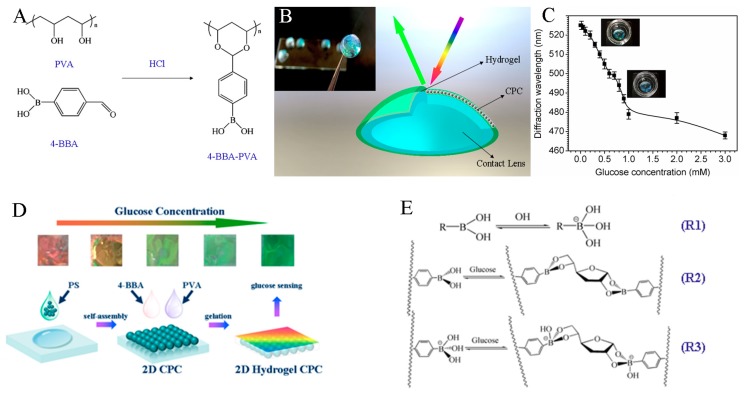
(**A**) Diagram and photograph (insert) of a hydrogel CPC sensing lens [89]. (**B**) The diffraction response at low glucose concentration (insert is the photograph of the diffraction-color-changeable lens sample). (**C**) Reaction between polyvinyl alcohol (PVA) and 4-BBA [90]. (**D**) Illustration of the construction of the PVA hydrogel CPC; the photographs show the forward-diffraction color change from red, through yellow, to green [91]. (**E**) Scheme of equilibrium between boronic acid and glucose in dilute solution: boric acid and borate ions can be formed (R1), and boronic acid can reversibly bind glucose both in its neutral trigonal form (R2) and its charged tetrahedral form (R3).

Lenses, usually used for vision correction as a portable and accessible device, with its favorable biocompatibility with decades of clinical use, have now caught much attention in drug delivery and the tear analyte detection field [87,88]. Chen developed a series of glucose-sensitive hydrogel-based colloidal photonic crystal (CPC) materials [89,90,91]. Firstly, a hydrogel was physically crosslinked from the PVA/PEG system, which could offer an anti-fouling layer for the penetration of glucose, and then modified with 4-boronobenzaldehyde (4-BBA) (Figure 8A), improving the sensitivity for glucose [89]. A prototype of such a lens is shown in Figure 8B. Figure 8C states that the diffraction wavelength shifts at a relatively low glucose concentration. In the range from 0 to 1 mM covering tear glucose concentration, there is an approximate linear correlation between glucose concentration and the diffraction wavelength. This response is due to the acidic nature of boronic acid. With exposure to sugars, such as glucose, the chemistry of the boronic acid moiety is changed. Boronic acid can generate protons by abstracting a hydroxide unit from water [90]. On the other hand, two-dimensional (2D) CPC glucose sensors, which are monolayered architectures of colloidal particles, were developed as shown in Figure 8D. The mechanism of phenylboronic molecular reaction with glucose involves (Figure 8E) (1) boronic acid binding to glucose in its neutral trigonal form; (2) hydroxylation of phenylboronic molecules in alkaline solution, and OH^−^ attacking the B atom, leading to a conformation transition of phenylboronic molecule into a tetrahedral anion; (3) the formed tetrahedral anion of phenylboronic molecules combining with carbohydrate molecules to form five-membered or six-membered cyclic complexes, which can be hydrolyzed in acidic solutions to recover boronic acid compounds and carbohydrate molecules. Therefore, the response of the whole sensing process is reversible [91].

In the field of electrochemistry, Jing has prepared a series of flexible sensors with good biocompatibility, super-long stretchability, self-adherence, and self-repairing performance [92]. Dopamine has good biocompatibility and can be used to reduce graphene oxide and improve its dispersibility in hydrogel solutions. The conductivity of injectable and self-repairing composite hydrogels can reach 1.22 mS/cm, and the beating frequency of myocardial cells in the hydrogel extract can reach 54 beats per minute [93].

### 4.5. Drug Delivery and Release

**Figure 9:**
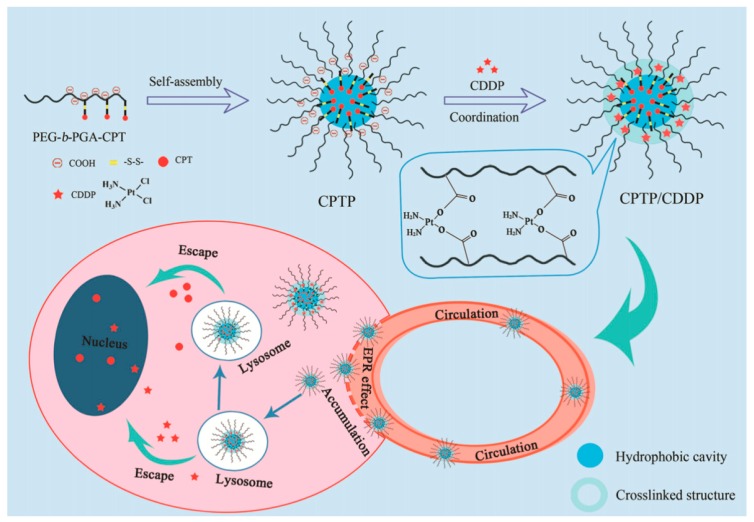
Schematic illustration of dual stable nanomedicines constructed by cisplatin-crosslinked camptothecin prodrug micelles for effective drug delivery [94].

Nanodrugs have great potential and application prospects in the clinical treatment of cancer. Nanomedicine is generally composed of carriers and chemotherapeutic drugs. Drugs play a therapeutic role, and carriers play an embedding and transporting role. Polymer micelles have attracted much attention in recent years because of their good biocompatibility, easy structure modification, and “core-shell” structure. Li constructed cisplatin-crosslinked polymer prodrug micelle nanodrugs with a dual stabilization effect by coordination complexation and synergistic stabilization of polymer prodrugs [94]. Figure 9 shows the design and preparation of polyethylene glycol-polyglutamic acid (PEG–b–PGA) with good biocompatibility as the main polymer chain and the camptothecin (CPT) precursor with a disulfide bond as the linker. The hydrophobic properties of CPT were used to construct the polymer precursor micelles. Finally, the polymer precursor micelles with double stability were constructed by complexing carboxyl groups with cisplatin (CDDP). The results show that the nanodrug can significantly improve the retention time in blood circulation and achieve effective accumulation in tumor lesions, showing excellent anti-tumor effect, and has good application prospects.

**Figure 10:**
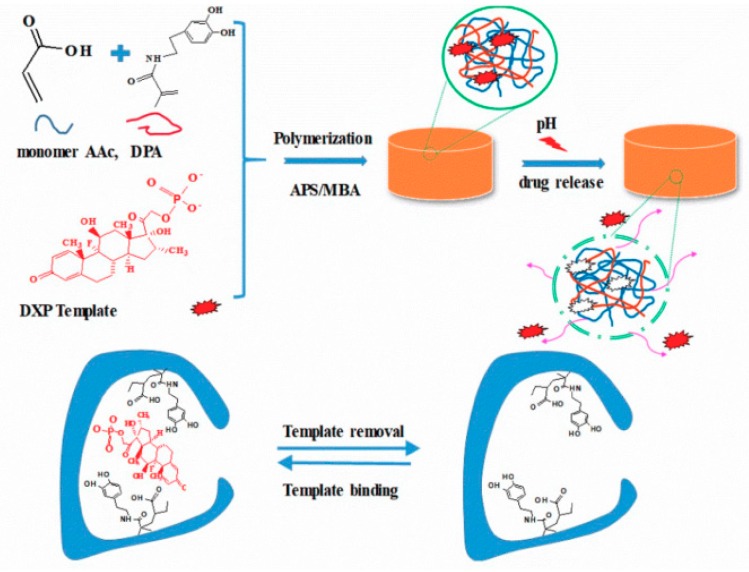
The schematic diagram of the preparation of pH-responsive molecularly imprinted hydrogels [95].

Wei prepared a novel pH-responsive molecularly imprinted polymer hydrogel that can effectively control the release of dexamethasone sodium phosphate (DXP) [95]. As shown in Figure 10, molecularly imprinted polymer (MIP) hydrogels were prepared by precipitation polymerization using dopamine-acrylamide (DPA) and acrylic acid (AAc) as functional monomers and DXP as template molecules. The results showed that the drug loading rate of MIP hydrogels was significantly higher than that of non-molecularly imprinted polymer (NIP) hydrogels in different DXP solutions. MIP hydrogels release only 58% of the drug in 24 h, while NIP hydrogels release more than 80% of the drug in 24 h. This controlled slow-release property of MIP hydrogels lasts for seven days. In addition, DXP-imprinted hydrogels exhibit pH-dependent properties due to the molecular interaction between the polymerized DPA and AAc.

## 5. Conclusions

The high water content and void structure of the hydrogels has resulted in their limited application. By increasing the polymer concentration, the mechanical strength can be increased with poor biocompatibility and lower degradability, and this is the main contradiction in the application of mechanical properties of hydrogels in the biomedical field.

Research progress in the past two years has demonstrated that the introduction of non-chemical bonds into the system has become an effective method of improving the strength of hydrogels, but the synergistic effect between non-chemical bonds and chemical bonds still need further research and development. The development of hydrogels with special physical properties, which are based on the premise of ensuring good biocompatibility and biodegradability, should be more similar to bio-gels in terms of onlooker structure and biological function.

High-end medicine is one of the potential applications for high-intensity hydrogels, such as human tissue repair, organ culture in vitro, and targeted cancer treatment. With further intersection of polymer and biomedical science, attempts to give hydrogels unique physical properties through different methods are expected to be more widely used in the biomedical field and to achieve medical advancement.

## Figures and Tables

**Table 1:** Mechanical properties and examples of several hydrogels [12].

Materials	Examples	Tensile Strength	Tensile Modulus	Compressive Strength	Compressive Modulus
Traditional hydrogel [13,38]	PVA, PEG	1~100 kPa	<100 kPa	10~100 kPa	1~100 kPa
Double network hydrogel [39,40]	Agar/PAM	10 MPa	1 MPa	60 MPa	100 kPa
Nanocomposite hydrogel [22,41,42]	PEG/clay	255 kPa	16 kPa	3.7 MPa	38 kPa
Macromolecular Microsphere Composite Gel [43]	MMS–AAm	540kPa	270 kPa	78.6 MPa	--
Tetra-PEG Gel [44,45]	PEG–NH_2_/PEG–COOH	200 kPa	90 kPa	27 MPa	100 kPa
Topological Gel [34,46]	Polyrotaxane	20 kPa	--	--	350 kPa
Cartilage [47,48]	--	~3 MPa	~9 MPa	~35 MPa	~15 MPa
Collagen Fiber [13,49]	--	~75 MPa	~1000 MPa	--	--
Ligament [50,51]	--	~16 MPa	~250 MPa	--	--
